# A Study on Epidemic Information Screening, Prevention and Control of Public Opinion Based on Health and Medical Big Data: A Case Study of COVID-19

**DOI:** 10.3390/ijerph19169819

**Published:** 2022-08-09

**Authors:** Jinhai Li, Yunlei Ma, Xinglong Xu, Jiaming Pei, Youshi He

**Affiliations:** 1 College of Information Engineering, Taizhou University, Taizhou 225300, China; 2 Department of Personnel, Taizhou University, Taizhou 225300, China; 3 School of Management, Jiangsu University, Zhenjiang 212013, China; 4 School of Computer Science, The University of Sydney, Camperdown, NSW 2006, Australia

**Keywords:** health and medical big data, epidemic, early warning, COVID-19, public opinion, xgboost, subject matrix

## Abstract

The outbreak of the coronavirus disease 2019 (COVID-19) represents an alert for epidemic prevention and control in public health. Offline anti-epidemic work is the main battlefield of epidemic prevention and control. However, online epidemic information prevention and control cannot be ignored. The aim of this study was to identify reliable information sources and false epidemic information, as well as early warnings of public opinion about epidemic information that may affect social stability and endanger the people’s lives and property. Based on the analysis of health and medical big data, epidemic information screening and public opinion prevention and control research were decomposed into two modules. Eight characteristics were extracted from the four levels of coarse granularity, fine granularity, emotional tendency, and publisher behavior, and another regulatory feature was added, to build a false epidemic information identification model. Five early warning indicators of public opinion were selected from the macro level and the micro level to construct the early warning model of public opinion about epidemic information. Finally, an empirical analysis on COVID-19 information was conducted using big data analysis technology.

## 1. Introduction

At the beginning of 2020, the coronavirus (COVID-19) epidemic broke out. In line with the orderly implementation of offline anti-epidemic work, online anti-epidemic work has also been carried out. For the first time, the whole country was alerted about the local and national epidemic situation, while popularizing epidemic prevention measures and precautions to the public through the network platform. However, unlike offline anti-epidemic work, which has clear responsibilities and procedures, online anti-epidemic work focuses more on the massive amount of epidemic information released by various platforms and netizens, in addition to the official media. The massive amount of epidemic information makes it impossible for the public to choose channels to obtain epidemic information in the first place, and this information contains considerable quantities of false and unverified information. This may cause part of the public to receive the wrong information, indirectly at first. For example, information about false mask sales, false epidemic prevention measures, and unconfirmed information about patients led to social panic, and even caused the loss of people’s lives and property.

Therefore, identifying reliable online sources of information about the epidemic is critical. While identifying reliable sources of information, it is crucial to identify the authenticity of the epidemic information itself. On the basis of reliable information sources, it is necessary to identify the authenticity of the epidemic information and automatically filter out false epidemic information, to ensure that the public can obtain real and effective epidemic information. At the same time, real-time tracking of false epidemic information, early warning of public opinion about the epidemic information that may affect social stability and endanger the safety of people’s lives and property, as well as timely prevention and control measures, are needed.

Traditional public opinion early warning lies in the collection of webpage information from designated sources and the analysis and statistics of keywords [1]. However, the public opinion information of epidemics is different from the general network information. The epidemic information involves professional fields, such as health care, which are not simply capable of the public opinion analysis of general network information. This needs to be combined with professional knowledge of health care and other professional fields, to carry out public opinion analysis.

Therefore, network epidemic information data cannot be simply regarded as network data and are an important component of health and medical big data. With the introduction of health and medical big data policies in various countries, the application of health and medical big data has a guidance function. Health and medical big data will be a key combination in the field of medical public health and big data analysis in the future, while also providing a method for the screening of network epidemic information and public opinion prevention and control.

## 2. Theoretical Analysis

### 2.1. Health and Medical Big Data Analysis

In late 2014 and early 2015, *Science* magazine published two articles, “Public Health meets Big Data” [2] and “Integrating Big Data into public health system” [3], which promoted the application of big data in public health. As an extremely important part of big data [4], health and medical big data includes not only public health data, but also medical data and personal health data [5]. Health and medical big data are one of the most important basic strategic resources of a country. The integration, reorganization, research, mining, and utilization of health and medical big data conform to the policy requirements of the application and development of health and medical big data. Additionally, it is an inevitable trend to use big data as a decision-making tool.

Utilizing health and medical big data can provide individual and comprehensive pathological diagnoses and provide the basis for each patient to have the optimal clinical treatment plan [3,6]. Based on health and medical big data, gene sequencing technology is used to surpass the passive mode of traditional medicine and realize personalized medical treatment [7,8]. Moreover, health and medical big data can also analyze the demand trends of the public for drugs, determine a more efficient input–output ratio, and reasonably allocate resources. For example, during the outbreak of COVID-19, the government coordinated the allocation of medical resources, such as national masks, based on the analysis of health and medical big data.

Using health and medical big data to mine knowledge and promote innovation has become a new approach for medical decision-making and personal health management, while also providing scientific support for epidemic prevention and control. However, it deepens the threat to individual health information security and privacy [9].

### 2.2. Studies Related to COVID-19

Since the outbreak of COVID-19, scholars have performed a lot of research on COVID-19, from many perspectives. Including the source of COVID-19, the prevention and treatment of COVID-19, the impact of COVID-19 on society, and relevant analysis of big data. This section will focus on the analysis of COVID-19 related to health and medical big data and social impact.

Basile et al. took Reddit as their research object, considered structural variations in social posting behavior and emotional reactions based on the Plutchik model of basic emotions and unconventional emotions, and also studied the specific reactions of social posting during the pandemic versus normal periods [10]. Eraso et al. studied intentional and unintentional non-adherence to social distancing measures during COVID-19 and put forward suggestions for improvement [11].

Aebi et al. found that big data has the potential to strengthen our mental health prevention systems in the context of global public health crises. However, the application of big data in this aspect faces ethical and technical challenges [12]. Lee studied the correlation between COVID-19 sentiment and 11 select sector indices of the US stock market through the Daily News Sentiment Index and Google Trends data on coronavirus-related searches, and proposed strategic investment planning considering time lags, by visualizing the change of correlation level using time lag differences [13].

### 2.3. Identification and Analysis of False Information

The purpose of identifying and analyzing false information is to identify and filter out false network information and reserve real and useful network information for the later analysis of network information data [14]. The identification and analysis of false information can, not only reduce the size of the dataset to be analyzed, but it can also improve the accuracy of data analysis. The proportion of possible false data contained in a dataset will be reduced and the impact on the results of the data analysis will be reduced. Therefore, the identification and analysis of false information in health and medical big data can improve the accuracy of epidemic public opinion analysis based on network health and medical big data.

The function of identifying false information and filtering is similar to the process of identifying and filtering data noise in data analysis preprocessing, but this processing should occur prior to the data preprocessing.

At present, the identification and analysis of false comments [15] rarely involves the identification of false network health and medical big data. Network health and medical big data have distinct characteristics in the professional field. Therefore, on the basis of general false network information identification, it is necessary to combine the characteristics of network health and medical big data. Most of the public has a strong ability to distinguish false network information, such as false e-mails and false webpages. However, false network health and medical big data have strong camouflaging abilities, and it is difficult for people without professional health and medical knowledge to make an identification the first time. Due to uniqueness of the network health and medical big data, the research on false information identification for network health and medical big data has particular characteristics.

The design of false feature identification methods is the key step in false information identification [16]. In the early stage of research on false information identification, scholars focused on the authenticity of the information content itself and extracted features from the information content itself to identify false information [17]. However, identification accuracy is greatly affected by the category of informational content. Therefore, scholars began to pay more attention to the impact of publishers on the authenticity of information and judged the authenticity of information by analyzing the publishers’ behavioral characteristics [18]. In addition to the characteristics of the information itself and the publisher information, some scholars studied the identification of false information based on graph theory, which is characterized by the network relationship between three subjects: information, the information publisher, and the information description object [19]. Taking the characteristics of mutual relations between different subjects as a perfection of the characteristics of subjects, it is widely used in the identification of false publishers and groups, which provides a new way of identifying false information.

After the false feature design is completed, the identification method is designed, constructing the false information identification model and making the final judgment on information classification. According to the dependence of the identification method on the annotated data, the design of the identification method can be divided into three categories: using annotated data, using partially annotated data, and not using annotated data [16]. Among them, the most common method of identification is the use of annotated data to classify information into true and false categories, based on supervised machine learning text classification [20].

### 2.4. Early Warning Analysis of Online Public Opinion

The research on online public opinion mainly focuses on the characteristics of online public opinions, early warning of online public opinions, the influence of online public opinion, and the guidance of online public opinion.

Scholars have conducted in-depth analysis on the structure, communication, and subject characteristics of online public opinion, laying the foundation for the accurate expression of online public opinion, while also searching for more effective online public opinion monitoring methods and concepts [21].

Research on online public opinion early warning is the focus of research on online public opinion. The current mainstream research conception of online public opinion early warning is to establish an early warning index system through the collection and processing of network data, and to predict possible online public opinion events [22] by adopting Bayesian network modeling, gray prediction methods, system dynamics, and other methods [23].

In the research of online public opinion early warning, opinion mining is a key research content. Opinion mining is an important application of text mining. The emotional tendencies of text can be mined through opinion mining, which is one of the important indicators of online public opinion early warning. Opinion mining and text mining have been widely studied in many fields, in addition to the traditional machine learning methods, such as naive Bayes, support vector machine, maximum entropy, decision tree, etc. In recent years, deep learning technology has been widely used in opinion mining and text mining. For example, in the field of e-commerce, Da’u et al. proposed a recommendation system based on opinion mining. The opinion mining model was based on a multichannel deep convolutional neural network. The results showed that the accuracy of the recommender system was improved [24]. In the field of medicine, Fatiha et al. proposed a framework of opinion classification of drug reviews, and compared the accuracy of the opinions classification model of drug reviews based on CNN and RNN [25]. In the field of online public opinion, Kim et al. proposed an online public opinion evaluation method based on deep learning. The method generated time-series data on online sentiments toward the South Korean president, and compared this to traditional sentiment. The results demonstrated the independence of the masses’ online discourse [26].

The influence of online public opinion is greatly magnified by the rapid development and wide popularization of network technology. When an emergency occurs, online public opinion can, not only provide a reference basis for relevant government departments to deal with it, but it can also pose a risk of causing social problems if improperly guided [27].

Online public opinion has become an important factor affecting national interests and social stability. If we do not pay timely attention and correctly guide online public opinion, it will cause immeasurable impacts and losses to the economy and society. Based on the theories of communication, social psychology, and public management, many scholars have conducted comprehensive and in-depth discussions on the guidance of online public opinion, including aspects of the research and judgment system, guidance mechanisms, guidance groups, and improvement of guidance ability [28].

At present, online public opinion early warning pays more attention to the early warning of hot social issues. In the field of health and medicine, early warning research focuses on medical risks [29], mental health early warning [30], and physical health status early warning based on personal health and medical big data [31], while less attention is given to public opinion early warning research based on network health and medical big data.

To sum up, only real health and medical big data can correctly reflect epidemic information. However, most network data are not screened and filtered, and strict checks are lacking. Therefore, it is very important to identify reliable information sources [32]. However, health and medical big data from reliable sources may also contain a small amount of untrue data. Epidemic information is different from general network information and cannot tolerate any ambiguity. Further identification of the network health and medical big data, itself, remains a must.

The novelty of this study is that before the application of traditional methods for identifying false information, reliable information sources are first identified, which will greatly reduce the size of the dataset to be identified.

## 3. Analysis and Construction of the Model of Screening Epidemic Information, as Well as Public Opinion Prevention and Control Based on Health and Medical Big Data

### 3.1. Construction of the Identification Model for False Epidemic Information

#### 3.1.1. Extraction of Identification Features

The purpose of false epidemic information identification is to distinguish the true and false epidemic information from the massive network health and medical big data, as well as to filter out that false epidemic information, to ensure the accuracy of public opinion warning about the epidemic information. In fact, the identification of true and false epidemic information is a classification problem. For the classifier, it is necessary to extract suitable classification features to train the classification model and find the correct classification mode. Therefore, effective extraction of classification features of epidemic information is the key to identifying the authenticity of epidemic information. By combing the existing literature it was found that, in many research results, language features (such as emotional intensity and the number of feature words), text features (such as the number of clauses and the effective length of information), and the features of the publisher (such as the name of the publisher, the rank of the publisher, and the information released by the publisher) proved to be the most effective features [20,33]. On the basis of the existing research on identifying network spam information and extracting the features of the information itself, the features of the information publisher, this paper takes into account the domain specialization of the epidemic information and increases the number of subject features of epidemic information. It also considers that the current network environment is the era of the “we media” era, where everyone is an information publisher, while epidemic information has obvious health and medical specialty knowledge. Therefore, the information publisher behavior features include a feature of the publisher’s history of false information release. Based on the different feature levels, the above features are divided into four levels according to the feature granularity and type.

In summary, this paper extracts four kinds of classification features and one regulation feature, which are defined as follows:

(1) The features of the information subject extracted from the coarse granularity level are used to determine whether the information is relevant to the current epidemic theme.
(2) Text structure features extracted from the fine granularity level are used to determine whether the information is repetitive, irrelevant, or advertising.
(3) The emotional polarity extracted from the emotional orientation level is used to judge whether the information is overly complimentary or belittling.
(4) The features of publisher behavior extracted from the of publisher behavior level are used to judge whether the publisher is an authoritative person.
(5) The adjustment features extracted from the historical release of false information of a publisher are used to judge the possibility of the publisher releasing false information.

Based on the research results of existing scholars and the domain features of epidemic information, this paper extracted eight features from four levels: coarse granularity level, fine granularity level, emotional orientation level, and publisher behavior level, as shown in Table 1.

#### 3.1.2. Analysis and Calculation of the Identification Features

Subject relevance was calculated by using m nouns $N_{i}=\left[n_{1},n_{2},\ldots ,n_{m}\right]$ extracted from information *i* and the set epidemic subject feature words $F=\left[f_{1},f_{2},\ldots ,f_{k}\right]$ through a word co-occurrence calculation method, based on unsupervised learning point mutual information PMI [34]. We can calculate that:

(1) $$PMI\left(N,F\right)=\log\frac{p\left(N,F\right)}{p\left(N\right)p\left(F\right)}=\log\frac{p\left(N\left|F\right.\right)}{p\left(N\right)}=\log\frac{p\left(F\left|N\right.\right)}{p\left(F\right)}$$

Through Formula (1), we can calculate the relevance between each piece of information collected and the epidemic topic. The more relevant the subject is, the more likely the information is to be accurate.

The number of feature words refers to *W_i_*, the sum of the number of all nouns, verbs, adjectives, and adverbs in information *i*. These words are all effective words used to describe information. The more words there are, the more complete the description of information will be, and the more likely it is real information.

The number of clauses is *S_i_*, the sum of the number of all clauses in information *i*. The more clauses contained in the information, the more complete the structure of the information, and the more likely that the information will be true.

The effective length of information is obtained by the ratio *E_i_/T_i_*. *T_i_* is the total number of information characters of information *i*. *E_i_* is the number of characters after removing invalid characters from information *i*. The higher the ratio, the smaller the proportion of invalid characters contained in the information, and the greater the possibility of true information.

Emotional intensity is calculated by the proportion *P_i_*/(*P_i_* + *R_i_*) of the number of positive evaluation clauses *P_i_* in the sum of the number of positive evaluation clauses *P_i_* and negative evaluation clauses *R_i_* in information *i*. When the proportion of positive evaluation clauses is too large or too small, this means that the information is overly complimentary or belittling, and the possibility of the information being true is decreased.

The publisher’s name is set according to whether it is a real name. If it is a real name, the characteristic value is set to 1; otherwise, it is set to 0.

The level of a publisher is determined by the level of the platform where the publisher is located, and the level of the publisher can also reflect the authority of the publisher to some extent. Due to the inconsistency of the user rating systems of each platform, we have used a unified standard treatment and normalized it to three levels of 1 to 3, representing primary, intermediate, and advanced, respectively.

The amount of information released by the publisher *H_i_* is determined by the amount of information released by the publisher in their history. If the publisher has not published an information history, or published very little information, then the publisher may be temporarily registered, and the authenticity of the information cannot be guaranteed.

The publishing of historical false information is a regulating probability, which is set to 0 at the beginning. If false information is identified later, the probability will be increased. To speed up the algorithm implementation, when the probability reaches a certain threshold, θ, all the information released by the publisher will be gradually eliminated.

#### 3.1.3. Identification of False Epidemic Information

After calculating the feature value of all epidemic information, the next step is to identify and classify false epidemic information based on the feature value of the epidemic information.

Among the many classification algorithms, the XGBoost algorithm was selected in this paper. XGBoost is an ensemble learning algorithm and a tool for large-scale parallel boosting tree [35]. It is an improved version of the GBDT algorithm with a high efficiency. At the same time, XGBoost can rank the importance of each characteristic variable for the model output, which facilitates the selection of the model structure and improves the prediction accuracy. Therefore, XGBoost is more suitable for identifying multi-feature false epidemic information.

XGBoost is an additive model consisting of *k* base models. When the tree model to be trained in iteration *T* is *f_t_*(*x*), then the predicted value of sample *i* after iteration *t* is:

(2) $${\hat{y}}_{i}^{t}={\hat{y}}_{i}^{t-1}+f_{t}\left(x_{i}\right)$$

where ${\hat{y}}_{i}^{t-1}$ is the predicted result of iteration *t* − 1.

The objective function of XGBoost is composed of the loss function *l* of the model and the regularization term Ω that suppresses the model complexity. The objective function is defined as:

(3) $$Obj^{t}=\sum_{i=1}^{n}l\left(y_{i},{\hat{y}}_{i}\right)+\sum_{i=1}^{t}\Omega \left(f_{i}\right)$$

where $\sum_{i=1}^{n}l\left(y_{i},{\hat{y}}_{i}\right)$ is the deviation between the true value and the predicted value obtained by the loss function *l*. $\sum_{i=1}^{t}\Omega \left(f_{i}\right)$ is the sum of complexity of all *t* trees. It is added to the objective function as a regularization term to prevent overfitting of the model.

The approximate value of the objective function is obtained by expanding the objective function through Taylor’s formula and removing the constant, which has no influence on the optimization of the objective function:

(4) $$Obj^{t}=\sum_{i=1}^{n}\left[g_{i}f_{t}\left(x_{i}\right)+\frac{1}{2}h_{i}f_{t}^{2}\left(x_{i}\right)\right]+\Omega \left(f_{t}\right)$$

where $g_{i}$ is the first derivative of the loss function and $h_{i}$ is the second derivative of the loss function. The first derivative and the second derivative here are both derivatives with respect to ${\hat{y}}_{i}^{t-1}$. ${\hat{y}}_{i}^{t-1}$ is the predicted result of iteration *t* − 1, which is known. By optimizing the objective function, we can obtain *f_t_*(*x*) for each step. The complexity of the tree in XGBoost is determined by the number *T* of leaf nodes of all the generated decision trees and the *L*_2_ modulo square of the vector composed of the weights of all the nodes. The final objective function is:

(5) $$\begin{array}{l} Obj^{t}=\sum_{i=1}^{n}\left[g_{i}f_{t}\left(x_{i}\right)+\frac{1}{2}h_{i}f_{t}^{2}\left(x_{i}\right)\right]+\Omega \left(f_{t}\right)\\ =\sum_{i=1}^{n}\left[g_{i}w_{q\left(x_{i}\right)}+\frac{1}{2}h_{i}w_{q\left(x_{i}\right)}^{2}\right]+\gamma T+\frac{1}{2}\lambda \sum_{J=1}^{T}w_{j}^{2}\\ =\sum_{j=1}^{T}\left[\left(\sum_{i\in I_{j}}g_{i}\right)w_{j}+\frac{1}{2}\left(\sum_{i\in I_{j}}h_{i}+\lambda \right)w_{j}^{2}\right]+\gamma T\\ =\sum_{j=1}^{T}\left[G_{j}w_{j}+\frac{1}{2}\left(H_{j}+\lambda \right)w_{j}^{2}\right]+\gamma T \end{array}$$

where $I_{j}=\left\{i\left|q\left(x_{i}\right)=j\right.\right\}$ represents all samples *x_i_* belonging to the *j*th leaf node. *G_j_* is the sum of the first partial derivatives of the samples contained in leaf node *j*. *H_j_* is the sum of the second partial derivatives of the samples contained in leaf node *j*.

**Figure 1:**
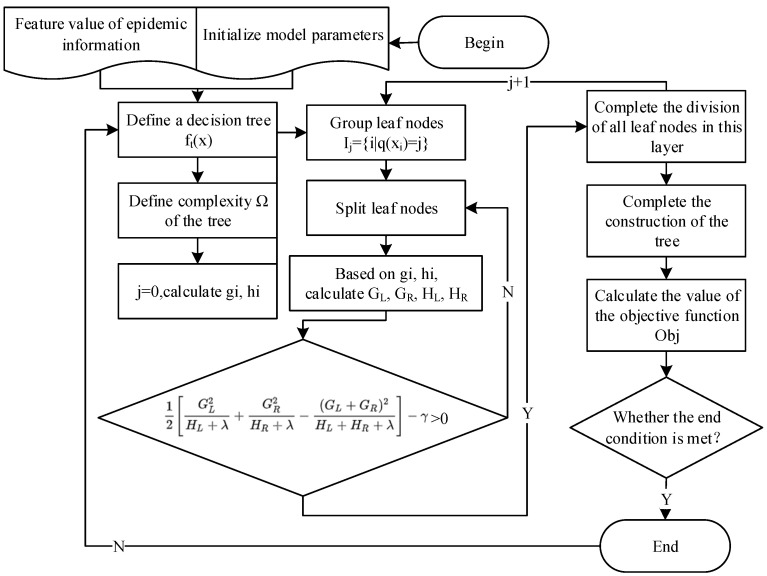
The identification process of fake epidemic information based on the XGBoost algorithm.

The identification process of fake epidemic information based on the XGBoost algorithm is shown in Figure 1.

**Figure 2:**
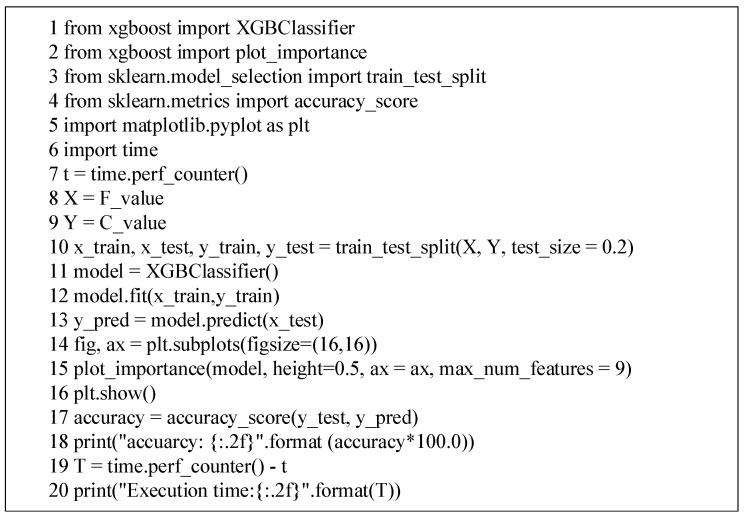
Implementation code of false epidemic information identification based on classifier XGBClassifier.

This paper used the XGBClassifier of the XGBoost classifier to train a certain number of pre marked sample sets, to obtain the classification model. Through this classification model, the collected epidemic information is identified, and then the information classified as false epidemic information by the classification model is filtered out. Part of the implementation code based on XGBClassifier is shown in Figure 2.

Where *F*_value is the dataset of the feature value of epidemic information from the sample set. The *C*_value is the dataset of false identification marks of epidemic information from the sample set.

### 3.2. The Establishment of an Epidemic Information Public Opinion Early Warning Model

#### 3.2.1. Process Analysis of Epidemic Information Public Opinion Early Warning

Through the false epidemic information identification model, we obtained effective data that can be used for epidemic information public opinion early warning analysis. The next step is to build an epidemic information public opinion early warning model; conduct public opinion early warning on the current epidemic information, to judge the current situation and future trend of the epidemic information public opinion; and conduct effective guidance, prevention, and control.

**Figure 3:**
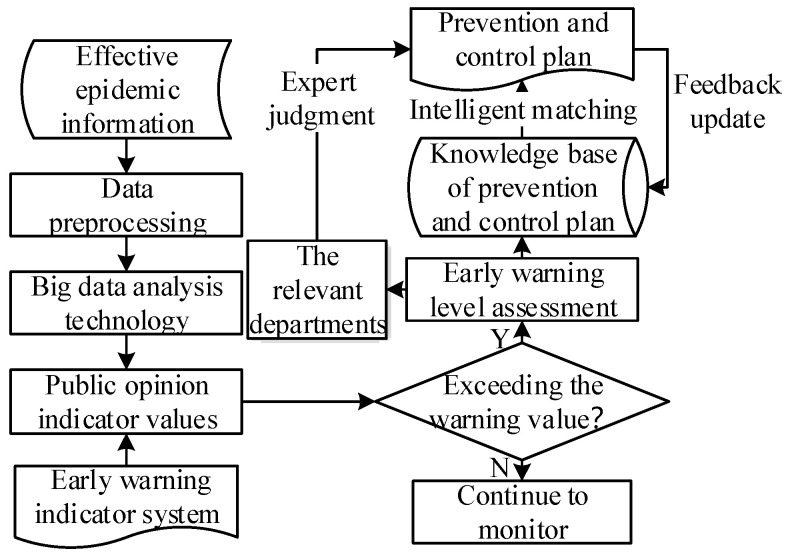
The specific working process of the epidemic information public opinion early warning model.

The working principle of the epidemic information public opinion early warning model is similar to the online public opinion early warning model for general emergencies [36]. However, it is more professional, in the setting of an early warning indicator system and prevention and control scheme. The workflow of the epidemic information public opinion early warning model is based on the input of effective epidemic information data and data analysis based on the early warning indicator system, to obtain the current epidemic information and public opinion indicator values. Through a comparative analysis with the early warning indicator system, the current public opinion situation is studied and judged, and the possible future public opinion trends are predicted. Public opinion prevention and control is carried out based on the plan in the knowledge base of prevention and control. The results of the data analysis are fed back to the relevant departments for scientific decision-making, and the scientific decisions formed by experts from relevant departments are updated to the public opinion prevention and control plan in the knowledge base, to ensure that the knowledge base is scientific, effective, and timely. The specific working process of the epidemic information public opinion early warning model is shown in Figure 3.

In the specific workflow of the epidemic information public opinion early warning model, the establishment of an early warning indicator system, and the big data analysis technology used to analyze the epidemic information public opinion, are two key processes.

#### 3.2.2. The Establishment of an Early Warning Indicator System

The establishment of the online public opinion early warning indicator system cannot be separated from certain established principles, which are closely related to the public opinion field [22]. The establishment of the epidemic information early warning indicator system also needs to follow the principles of early warning indicators in the field of health care:

(1) Early warning indicators should be representative, which can reflect the basic features of relevant indicators.
(2) Early warning indicators should be quantitative and can be numerically calculated according to big data analysis technology.
(3) Early warning indicators should have relevance, which can reflect the overall situation of early warning, based on the correlation between each early warning indicator.
(4) Early warning indicators should be forward-looking, reflecting the changes of a public opinion trends in the future, and benefit from earlier deployment.

Based on the above principles and considering the particularity of epidemic public opinion early warning, this paper selected two indicators from the macro level: the quantitative change rate of epidemic information, and the regional coverage rate of epidemic information. The paper also selects three indicators from the micro level: the emotional tendency of epidemic information, the concentration degree of the epidemic information subject, and the new subject regarding epidemic information. Specific descriptions of each indicator are shown in Table 2. The calculation method of each indicator based on big data analysis technology will be explained in detail in the empirical section.

## 4. Empirical Analysis of COVID-19 Data Based on Big Data Technology

### 4.1. Data Collection of Epidemic Information

With the rise of social media and online medical platforms, health and medical big data based on the internet have gradually become an important part of health and medical big data [37]. In contrast to previous health and medical big data sources, the research objects are mainly electronic medical records, medical images, public health data, and personal health data originating offline. Network health and medical big data mainly covers the search records of network users, social media users’ preferences, and online medical platforms, etc. Research on public opinion about epidemic information takes network health and medical big data as the research object. Among them, online user search records have been confirmed by domestic and international scholars to be effective for the research of network health and medical big data. The most influential study was that in which Hulth successfully predicted the outbreak of influenza by using search keywords from the Google search engine [38]. Professor Yuan Qingyu of the Chinese Academy of Medical Sciences analyzed influenza prediction based on Baidu search data [39]. Therefore, this paper takes Baidu search data as the data source for epidemic information public opinion analysis at this stage. In the future, the composition of data sources for epidemic information public opinion will be further expanded on this basis.

The data source is the webpages from the Baidu search engine, based on the keywords “epidemic “, “COVID-19”, and “virus”. Then, a requests module based on Python collects the top 500 webpages. Through webpage preprocessing for webpage cleaning, a transfer matrix, M, is constructed for the cleaned webpages, and the transfer matrix M records the chain in and chain out of all webpages.

### 4.2. Identification of Reliable Sources of Epidemic Data

The transfer matrix *M* constructed based on the 500 collected webpages is shown below.

$$M=\left|\begin{array}{cccccc} 0 & 0 & \ldots & 1/5 & \ldots & 0\\ 1/10 & 0 & \ldots & 1/8 & \ldots & 0\\ \ldots & \ldots & \ldots & \ldots & \ldots & \ldots \\ 1/10 & 0 & \ldots & 1/13 & \ldots & 0\\ \ldots & \ldots & \ldots & \ldots & \ldots & \ldots \\ 0 & 0 & \ldots & 1/16 & \ldots & 0 \end{array}\right|$$

where in the second row, the first column of the matrix *M*_21_ = 1/10 means that there are 10 outgoing links from the first webpage, and this links to the second page. Through the matrix *M*, we can find that if the value of each column is not 0, then the value must be equal. Matrix *M* is a 500 × 500 matrix, and the antepenultimate column in matrix *M* is not the real antepenultimate column. Thus, the value displayed in this column is not the outgoing link of the same webpage. A value that is 0 means that there are no outgoing links from the current webpage to the page. A value that is not 0 signifies the reciprocal of the number of outgoing links from the current page to other pages. The number of outgoing links on one webpage is a fixed value; obviously, the first column has a total of 10 1/10.

According to the reliability data source model constructed in reference [32], a total of 342 pages of reliable epidemic data sources were calculated, some of which are shown in Table 3.

### 4.3. Feature Extraction and Identification of False Epidemic Information

The requests module based on Python crawls the text information of these 342 webpages and saves all the text information in SQLite, the database of Python. Through the constructed identification model for false epidemic information, the saved epidemic text information was identified and filtered out.

The specific implementation process of the feature extraction and identification model of false epidemic information is as follows.

STEP 1. Calculation of feature *F*1: subject relevance.

Through this formula $PMI\left(N,F\right)=\log\frac{p\left(N,F\right)}{p\left(N\right)p\left(F\right)}=\log\frac{p\left(N\left|F\right.\right)}{p\left(N\right)}=\log\frac{p\left(F\left|N\right.\right)}{p\left(F\right)}$, the relevance between each piece of information collected and the epidemic topic is calculated.

STEP 2. Calculation of feature *F*2: number of feature words.

Calculate the sum *W_i_* of the number of all nouns, verbs, adjectives, and adverbs in information *i*.

STEP 3. Calculation of feature *F*3: number of clauses.

The number of clauses is *S_i_*, the sum of the number of all clauses in information *i*.

STEP 4. Calculation of feature *F*4: effective length of information.

The effective length of information is obtained using the ratio *E_i_/T_i_*. *T_i_* is the total number of information characters of information *i*. *E_i_* is the number of characters after removing invalid characters from information *i*.

STEP 5. Calculation of feature *F*5: emotional intensity.

The emotional intensity is calculated using the proportion *P_i_*/(*P_i_* + *R_i_*) of the number of positive evaluation clauses *P_i_* in the sum of the number of positive evaluation clauses *P_i_* and negative evaluation clauses *R_i_* in information *i*.

STEP 6. Calculation of feature *F*6: publisher’s name.

The publisher’s name is set according to whether it is a real name. If it is a real name, the characteristic value is set to 1; otherwise, it is set to 0.

STEP 7. Calculation of feature *F*7: level of publisher.

The level of the publisher is determined by the level of the platform where the publisher is located and normalized to three levels, from 1 to 3.

STEP 8. Calculation of feature *F*8: amount of information published by the publisher.

*H_i_* is determined by the amount of information released by the publisher over their history.

STEP 9. Calculation of feature *F*9: the historical release of false information by the publisher.

This is set to 0 at the beginning. If false information is identified later, the probability will be increased. To speed up the algorithm implementation, when the probability reaches a certain threshold θ = 0.5, all the information released by the publisher will be gradually eliminated.

STEP 10. The XGBClassifier of the XGBoost classifier is used to train the 80% pre marked sample sets and obtain the classification model.

STEP 11. The remaining 20% of the test set is used to test the trained classification model, and the accuracy and execution time of the model are obtained.

Finally, 258 effective pieces of epidemic information were obtained. Some of the epidemic text information is shown in Table 4.

Based on big data analysis technology, public opinion early warning was conducted on 258 effective pieces of epidemic information, to judge the current situation and the future trends of public opinion about the epidemic information, while carrying out effective guidance, prevention, and control. The results of the public opinion early warning analysis are visualized in the form of a data visualization below.

### 4.4. Visualization of Epidemic Information Public Opinion Early Warning

Through data visualization technology, we can make the dense epidemic public opinion early warning information more intuitive and easier to understand.

This paper selects two indicators from the macro level: the quantitative change rate of epidemic information, and the regional coverage rate of epidemic information. The paper also selects three indicators from the micro level: the emotional tendency of epidemic information, the concentration degree of epidemic information subject, and the new subject regarding epidemic information. We use two groups of epidemic information data, from 4 February 2020 to 12 February 2020, and from 20 March 2020 to 28 March 2020, as examples. The calculation process of each indicator of epidemic information public opinion early warning is as follows.

STEP 1. Calculation of indicator *I1*: change rate of epidemic information quantity.

The change rate of epidemic information quantity *Qr* is calculated from the adjacent unit time *t*1 to *t*2 using the ratio of the difference between the epidemic information data *l*_2_ at time *t*2 and the epidemic information data *l_1_* at time *t*1 to the epidemic information data *l*1: *Qr* = (*l*_2_ − l_1_)/*l*_1_.

STEP 2. Calculation of indicator *I*2: regional coverage rate of epidemic information.

The regional coverage of epidemic information, *Ac*, is calculated by combining all of the epidemic information into one complete text message and mining place words from the text. First, place names are clustered according to provincial administrative regions, and then the set of provincial administrative regions *p_l_* is compared, which is achieved after clustering with the set of provincial administrative regions of country *P*. Then, the regional coverage rate of epidemic information *Ac* = *p_l_*/*P* is calculated.

STEP 3. Calculation of indicator *I*3: emotional tendency of epidemic information.

Epidemic information emotional tendency *Et* can be obtained by synthesizing the emotional intensity of each piece of epidemic information, and the emotional intensity of each piece of epidemic information has been calculated at the stage of the identification of false epidemic information, which can be directly used here. Taking the average emotional intensity of all *n* effective epidemic information as the emotional tendency of epidemic information, according to the calculation method of emotional intensity in the false epidemic information identification model, the value range of emotional intensity is (0, 1):

(6) $$Et=\sum_{i=1}^{n}\frac{P_{i}}{\left(P_{i}+R_{i}\right)*n}$$

STEP 4. Calculation of indicator *I*4: concentration degree of epidemic information subject.

The concentration degree of the epidemic information subject *Tc* first represents each piece of epidemic information as a *t* subject list $\left[n_{1},n_{2},\ldots ,n_{m}\right]$, with all of the nouns contained in the information, and then combining all *n* subject lists into a subject matrix *Mt*:

$$Mt=\left|\begin{array}{cccc} n1_{1} & n1_{2} & \ldots & n1_{m}\\ n2_{1} & n2_{2} & \ldots & n2_{m}\\ \ldots & \ldots & \ldots & \ldots \\ nn_{1} & nn_{2} & \ldots & nn_{m} \end{array}\right|$$

The subject relevancy in the topic matrix *Mt* was calculated using point mutual information PMI, the subject relevancy used adaptive clustering, and the number of clusters c was obtained. The amount of epidemic information in each cluster reflects the degree of subject concentration of epidemic information *Tc* = *n*/*c*. The lesser the cluster, the higher the degree of subject concentration of epidemic information, and the cluster with the most epidemic information can be further analyzed to determine what subjects people focus on.

STEP 5. Calculation of indicator *I*5: new subject regarding epidemic information.

A new subject of epidemic information *Nt* is based on the clusters that were clustered previously. The subject relevance between the subject cluster of the epidemic information in the current time period and that of the previous time period can be compared and analyzed. The subject relevance between the two time clusters can be calculated using PMI. The cluster of current times with low subject relevance can be the new epidemic subject *Nt*.

**Figure 4:**
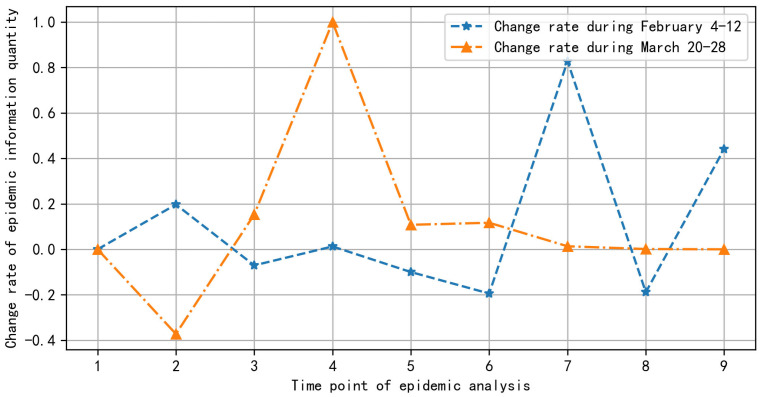
Change rate of epidemic information quantity.

After each indicator of epidemic information public opinion early warning has been calculated, first the data visualization is carried out on the two early warning indicators: the change rate of epidemic information quantity, and the regional coverage rate of epidemic information at the macro level. The visualization results of the change rate of epidemic information quantity are shown in Figure 4.

In Figure 4, the abscissa represents the day of the analysis period, and the ordinate represents the rate of change in the amount of epidemic information from the previous day. As seen in Figure 4, the changing rate of epidemic information quantity during February 4–12 increased greatly on the 7th and 9th days. By analyzing the epidemic information, we found that the 7th day was 10 February. The international expert group of the WHO investigated COVID-19 in China. The 9th day was 12 February. On the eve of that day, the director general of WHO, at the global forum on research and innovation in Geneva, announced that the disease caused by the new coronavirus would be officially named “COVID-19”. A large amount of information from the WHO epidemic situation report was released on the morning of 12 February.

**Figure 5:**
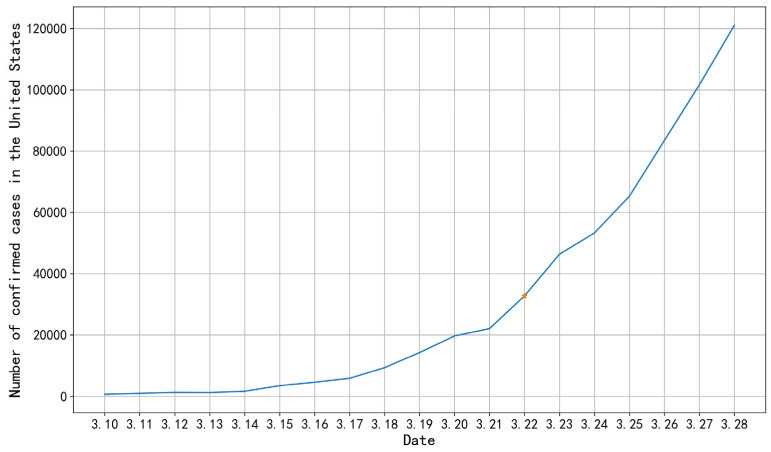
Number of confirmed cases in the United States from 10 March to 28.

The change rate of epidemic information quantity from 20 March to 28 increased substantially on the third and fourth days. By analyzing the epidemic information, we found that the epidemic situation in the United States became the global focus on Day 3 and Day 4, 22 March and 23. By collecting data on the number of confirmed cases in the United States from 10 March to 28, a change chart of the number of confirmed cases in the United States was drawn. From the change in the number of confirmed cases in the United States in Figure 5, it can be seen that the number of confirmed cases in the United States shows a straight-line upward trend from 22 March. In Figure 5, the abscissa represents the date, and the ordinate represents the number of confirmed cases.

**Figure 6:**
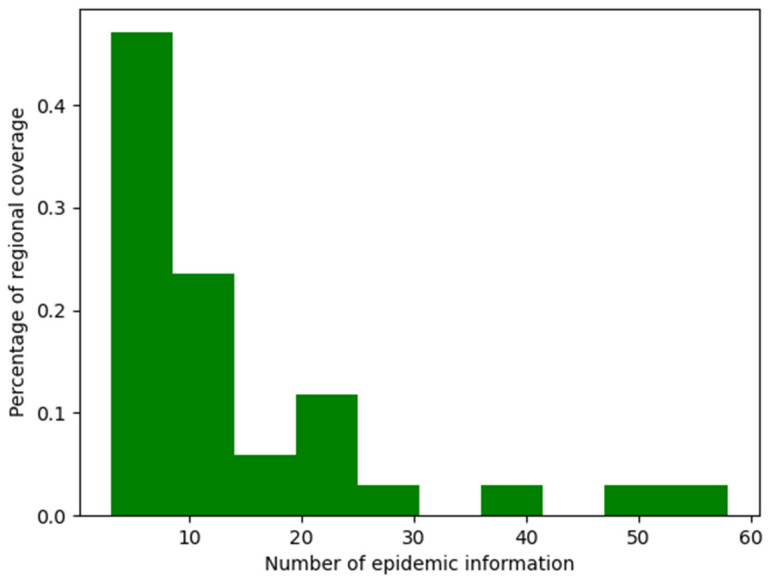
Regional coverage of epidemic information in China.

Taking China as an example, a visualization of the regional coverage data of epidemic information in China is shown in Figure 6. In Figure 6, the abscissa represents the amount of epidemic information, and the ordinate represents the coverage rate of the area involved in the epidemic information. As seen from the figure, all regions in China were involved in the epidemic information. This is because it was still the epidemic period, and epidemic prevention and control was still an important task for all regions in China. From the size of the abscissa of each bin, it was found that the extent of epidemic information in different regions of China was different. The larger the abscissa of the bin is, the more information about the epidemic situation in this area. From Figure 6 and after querying the associated data of the regional coverage rate of epidemic information, we found that the amount of pieces of epidemic information was more than 30 in Hubei, Beijing, and Shanghai. This means that these three areas needed to be focused on.

**Figure 7:**
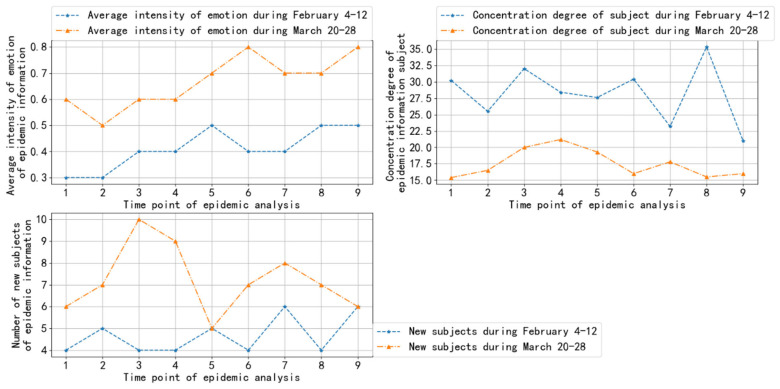
Public opinion indicator value at the micro level.

Next, we continued to visualize the three early warning indicators of emotional tendency of epidemic information, the concentration degree of epidemic information subject, and new subject regarding epidemic information, at the micro level. We used the two groups of epidemic information data collected from 4 February 2020 to 12 February 2020, and from 20 March 2020 to 28 March 2020 as examples. The visualization results are shown in Figure 7.

In Figure 7, the abscissa represents the day of the analysis period, and the ordinate represents the average intensity of emotion of epidemic information, the degree of concentration of the epidemic information subject, and the number of new subjects of epidemic information. From Figure 7, it is clear that the indicator value of emotional tendency of epidemic information from 20 March to 28 was considerably higher than that from 4 February to 12, and the average intensity of emotion was above 0.5, indicating that positive emotion was stronger than negative emotion. This was due to the success of epidemic prevention and control in China. China was basically in a stage of social order and stability from 20 March to 28 March, and public emotion was relatively positive.

The public opinion indicator value of the degree of concentration of the epidemic information subject from 20 March to 28 was considerably lower than that from 4 February to 12. This is because China was in the most difficult period of the epidemic from 4 February to 12. Public attention was focused on epidemic trends and epidemic prevention and control.

The indicator value of the new subject regarding epidemic information increased considerably from 20 March to 28 compared with 4 February to 12. It was found that the new subject regarding epidemic information had a significant negative correlation with the degree of concentration of the epidemic information subject. This is because the higher the concentration of the subject, the less likely a new subject will be generated. The indicator value of the new subject regarding epidemic information fluctuated substantially on the 7th and 9th days from 4 February to 12 and the 2nd and 3rd days from 20 March to 28, which was mainly related to the large increase in epidemic information on these days.

Through data visualization technology, the visualization display of the early warning indicator values of the epidemic information public opinion obtained by big data analysis technology can grasp the key points of the epidemic information public opinion prevention and control, the first time, and greatly reduce the preparation time for epidemic information public opinion prevention and control.

### 4.5. Evaluation of the Model Performance

The model performance was evaluated by comparing the accuracy and execution time of the model with the traditional models. Classical classification models were selected, including naive Bayes model, KNN model, and SVM model.

In addition, in order to verify the effectiveness of the identification features for false epidemic information selected in the paper, the model constructed in the paper was compared and analyzed with the model, without considering the features *F*1, subject relevance, and *F*9, the release of historical false information by the publisher.

**Figure 8:**
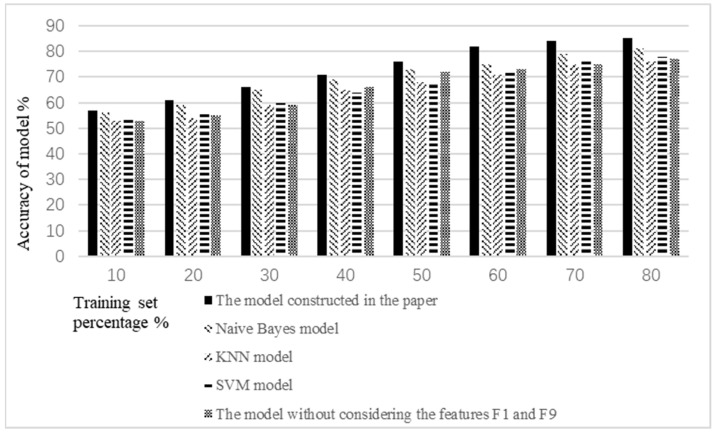
The accuracy comparison of the five models.

The accuracy comparison results are shown in Figure 8. As seen from Figure 8, the accuracy of the model constructed in the paper did not show obvious advantages while the percentage of the training set was less than 40%. This is because the model was not trained enough when the training set was small. With the increase of the percentage of the training set, i.e., when the percentage of the training set was greater than 40%, the accuracy of the model constructed in the paper was more accurate than the traditional classification models and the model without considering the features *F*1 and *F*9. This shows that the accuracy of the model constructed in the paper is better than that of the traditional classification models and the model without considering the features *F*1 and *F*9. In addition, it shows that the features *F*1, subject relevance, and *F*9, the release of historical false information by the publisher, have a significant impact on the accuracy of the model.

**Figure 9:**
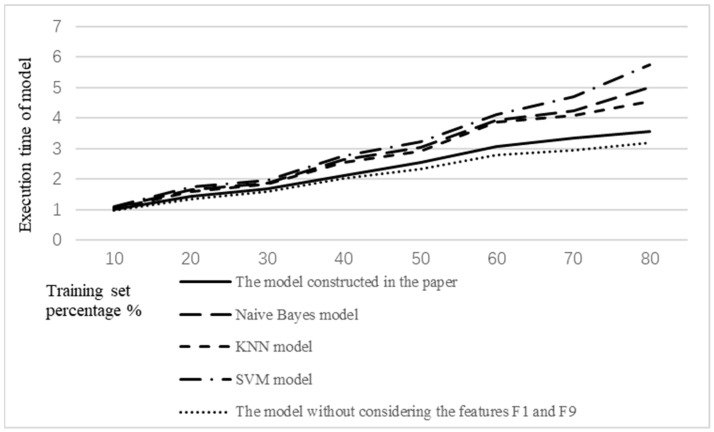
Execution time comparison of the five models.

In addition to the accuracy of the model, the efficiency of the model is also an important index to judge the performance of the model. The efficiency of the model was verified by comparison with the execution time of the traditional classification model and the model without considering the features *F*1 and *F*9. The execution time comparison of the five models is shown in Figure 9. At the same time, to facilitate the observation of a gap between the data, the execution time of the model constructed in the paper using 10% of the training set was set as standard 1, and the remaining execution times were standardized to this point.

As seen from Figure 9, although the execution time of the model constructed in the paper was lower than that of the traditional classification model, it did not show obvious advantages when the percentage of the training set was less than 60%. However, when the percentage of the training set was greater than 60%, the advantage was relatively obvious. This is because XGBoost, which is based on the model in the paper, is an ensemble learning algorithm. It has high efficiency. However, the execution time of the model constructed in the paper was increased compared with the model without considering the features *F*1 and *F*9. This is due to the reduction of training features, which reduces the amount of computation required. Therefore, the execution time of the model without considering features *F*1 and *F*9 has certain advantages over the model constructed in the paper. However, in the validation experiment of the accuracy of the model, we know that the accuracy of the model without considering features *F*1 and *F*9 was lower. Therefore, features *F*1 and *F*9 cannot be ignored.

## 5. Conclusions

Based on the domain knowledge of health and medical big data, as well as the author’s existing reliable data source research results, this paper conducted a detailed and in-depth study on the identification and filtering of false epidemic information, as well as the early warning of public opinion about epidemic information. Through the analysis of the false epidemic information identification model and the epidemic information public opinion early warning model, this paper expounds the basic idea of this study and the working principle and process of each model. We also made an empirical analysis of the relevant epidemic information based on the new coronavirus using big data analysis technology, and while carrying out a visualization display of the data. This paper analyzed the feasibility of epidemic information identification and public opinion prevention and control based on health and medical big data, from the two aspects of data support and calculation analysis support, which provide a new perspective for epidemic prevention and control research. This is also an objective demand for further strengthening the prevention and control of public health and major infectious diseases. Relevant departments can closely follow the public opinion trends of the epidemic situation with the help of early warning results, allowing timely guidance and avoiding public panic.

The false epidemic identification model constructed in the paper has certain advantages over the traditional classification model in terms of the accuracy and execution time of model. When the percentage of the training set reaches 80%, the accuracy of the model reaches 85%. Moreover, the accuracy of the model also becomes stable. However, similarly to the traditional model, the accuracy of the model is low when the amount of training is insufficient. In addition, under the premise of the same identification features, the model constructed in this paper has a higher efficiency than the traditional classification model. When the percentage of the training set reaches 80%, compared with the traditional model, the execution time of the model is reduced by about 30%. However, in order to increase the accuracy of the model, the identification features are increased accordingly, which also leads to a reduction of the efficiency of the model. How to strike a balance between the accuracy of the model and the efficiency of the model will also need to be focused on in the future.

At the same time, research on the feature extraction of false epidemic information identification and the establishment of an early warning indicator system for epidemic information public opinion is not sufficiently in-depth. In future, based on the existing research, the above problems will be expanded, and more scientific and reasonable feature extraction methods, along with public opinion early warning indicator systems, will be introduced, to improve false epidemic information identification and the reliability of public opinion early warning about epidemic information.

## Figures and Tables

**Table 1:** Identification features of false epidemic information.

Feature Level	Feature Number	Feature Identification
Coarse granularity	*F*1	Subject relevance
Fine granularity level	*F*2	Number of feature words
	*F*3	Number of clauses
	*F*4	Effective length of information
Emotional orientation	*F*5	Emotional intensity
Publisher behavior	*F*6	Publisher’s name
	*F*7	Level of publisher
	*F*8	Amount of information published by the publisher
Regulatory feature	*F*9	The release of historical false information by the publisher

**Table 2:** Early warning indicator system of epidemic information public opinion.

Indicator Hierarchy	Indicator Serial Number	Indicator Influencing Factors	Indicator Description
Macro level	*I*1	Change rate of epidemic information quantity	This indicator reflects the changing trend of the amount of epidemic information per unit of time. If the change rate is positive, this indicates that the number of people paying attention to the epidemic continues to increase; if the change rate is negative, this indicates that the number of people paying attention to the epidemic continues to decrease.
	*I*2	Regional coverage rate of epidemic information	This indicator reflects the regional distribution involved in the epidemic information. If the coverage rate is high, this indicates that the epidemic situation is a concern in many regions.
Micro level	*I*3	Emotional tendency of epidemic information	This indicator reflects the public’s attitude toward the epidemic situation. If the emotional tendency continues to increase, this indicates that the public has a positive attitude toward the epidemic situation.
	*I*4	Concentration degree of epidemic information subject	This indicator reflects the extent to which some subjects of the epidemic are a concern. If the concentration is high, this means that most people are more concerned about some aspect of the epidemic situation.
	*I*5	New subject regarding epidemic information	This indicator reflects a subject of epidemic information that has not been a concern before. If a new subject emerges, this means that the epidemic has changed and attracted wide public attention.

**Table 3:** Partial webpages of reliable epidemic data sources (accessed on 31 March 2020).

ID	URL
6	https://new.qq.com/omn/20200325/20200325A0C3ML00.html
32	http://dy.163.com/v2/article/detail/F8IH17IT0514WPGB.html
…	…

**Table 4:** Partial text information of effective epidemic information.

ID	URL
4	Will the spread of COVID-19 become “prolonged”...
156	The COVID-19 pandemic, scientists have imagined five ways it could end ...
…	…

## Data Availability

The data analyzed in this study are available by reasonable request to the corresponding author.
